# Encephalopathy as the Sentinel Sign of a Cortical Stroke in a Patient Infected With Coronavirus Disease-19 (COVID-19)

**DOI:** 10.7759/cureus.8121

**Published:** 2020-05-14

**Authors:** Smit Deliwala, Sarah Abdulhamid, Mohamed Faisal Abusalih, Mohammed M Al-Qasmi, Ghassan Bachuwa

**Affiliations:** 1 Internal Medicine, Hurley Medical Center, Michigan State University, Flint, USA; 2 Neurology, Hurley Medical Center, Michigan State University, Flint, USA

**Keywords:** coronavirus disease 2019, covid 19, sars-cov-2, cerebrovascular accidents, cva, coronavirus, mca, ischemic cva, ischemic stroke, stroke systems of care

## Abstract

The novel coronavirus has challenged medical systems worldwide to provide optimal medical care in the setting of limited resources. Although we are uncovering many facets of its disease spectrum, with rapidly emerging data, there is still limited knowledge of the sequelae of this infection, making treatment guidelines incomplete and resulting in serious unpredictable outcomes in patients at seemingly low risk, especially ones afflicted by neurological consequences. We present a case of a cortical stroke in a 31-year-old coronavirus disease-19 (COVID-19) positive female with otherwise no stroke risk factors. We noted a correlation between cytokine release, encephalopathy, and the onset of stroke symptoms. Patients with marked pro-thrombotic and inflammatory markers may benefit from closer neurological monitoring and thromboprophylaxis at therapeutic doses. The establishment of acute care pathways to manage critically ill patients with neurological consequences may reverse the suboptimal outcome trends seen during the pandemic.

## Introduction

Around the turn of 2019, a novel coronavirus was seen as the focal cause of a cluster of symptoms that began in Wuhan, China and spread rapidly across continents with the World Health Organization (WHO) declaring a Public Health Emergency of International Concern known as severe acute respiratory syndrome coronavirus 2 (SARS-CoV-2), or coronavirus disease-19 (COVID-19). As of April 7, the Centers for Disease Control (CDC) reported over 390,000 cases with over 10,000 deaths within the United States (US), and a cumulative incidence increasing from 8.3 to 418 cases per 100,000 persons [1]. Globally, over three million cases have been reported, with close to one million confirmed cases and 50,000 deaths in the US as of April 28, signifying a three-fold increase in cases over weeks [2]. Risk factors include existing co-morbidities while middle and older-aged adults are most affected, with the latter often displaying severe disease and higher mortality rates. Among its various presentations, neurological sequelae were seen in 36.4%, while 5.7% had acute cerebrovascular accidents (CVA) [3]. COVID-19 is known to have thrombotic complications in nearly 31% of critically ill patients [4]. Although a temporal relationship between COVID-19 patients and stroke incidence has not been parsed out, we present a case of a young patient with no risk factors who developed a severe COVID-19 infection and neurological sequelae. Short and long term outcomes in these patients are unknown [5]. We aim to strengthen the existing literature, explore the development of strokes in COVID-19 patients, and discuss strategies to identify patients at a higher risk to optimize resource allocation.

## Case presentation

**Figure 1:**
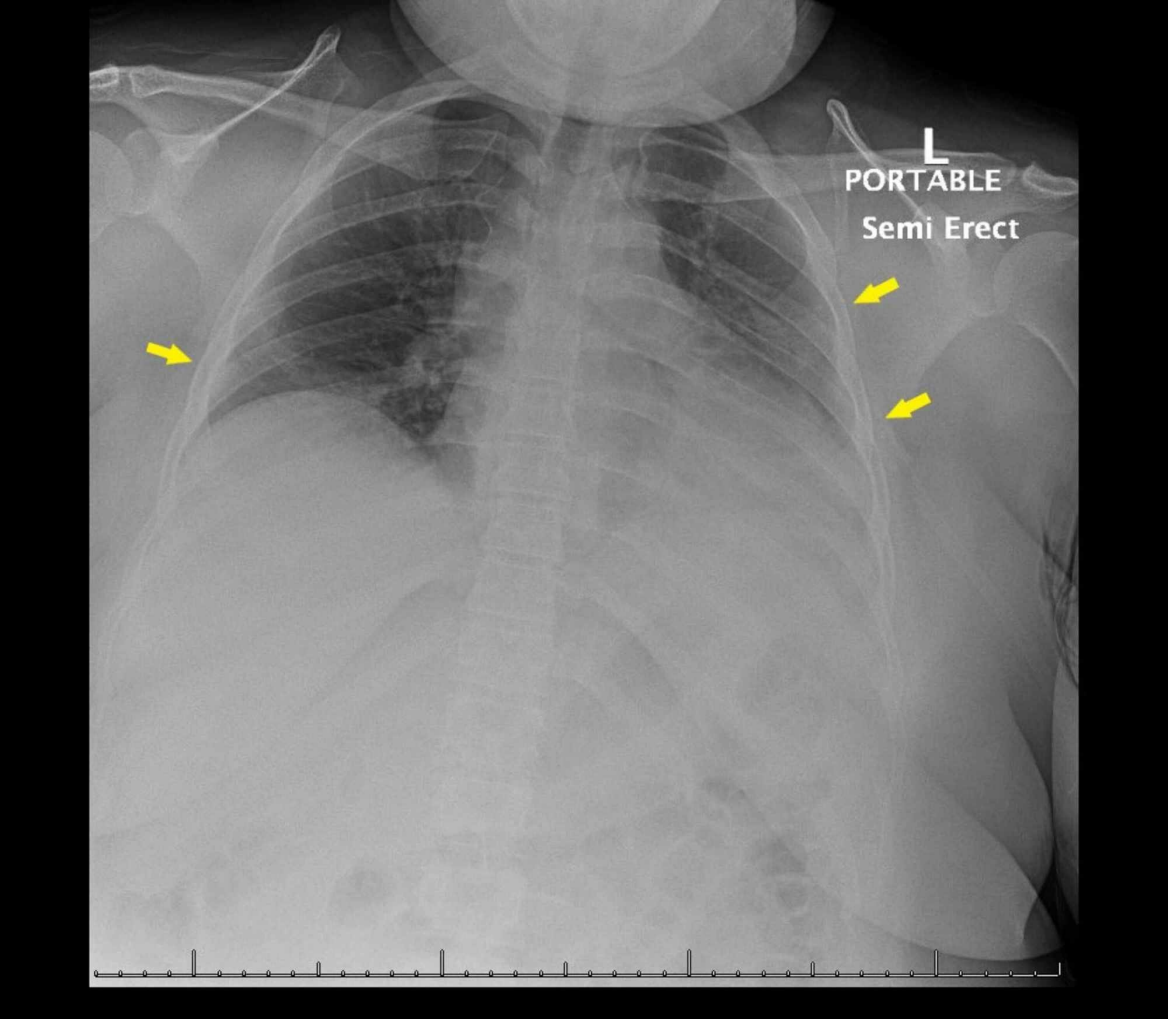
Initial chest radiograph with patchy opacities in the left mid to lower lung and right lung base suspicious for a multi-focal infectious process (arrows)

A previously healthy 31-year-old female presented to the emergency department (ED) with symptoms of fever, congestion, rhinorrhea, cough, myalgias, vomiting, and abdominal cramping evolving over five days. She worked at a factory, but was not exposed to any symptomatic contacts and did not use tobacco products, alcohol, or illicit substances. On arrival, her vitals were blood pressure - 97/73 mm Hg, heart rate - 127 beats/minute, respiratory rate - 18 breaths/minute while saturating was at 95% on supplemental oxygen, along with persistent fevers despite acetaminophen administration. On exam, she appeared ill, uncomfortable, and in acute distress. Initial investigations were remarkable for hypernatremia, acute kidney injury (AKI), and bilateral infiltrates on chest radiograph indicative of pneumonia (Figure 1). Ceftriaxone and azithromycin were started empirically with intravenous hydration, while nasopharyngeal swab for COVID-19 was obtained. Her hypoxia and fevers continued to worsen with increasing oxygen requirements, evolving hypotension, and tachypnea, requiring intubation and sedation with transfer to the intensive care unit (ICU).

**Figure 2:**
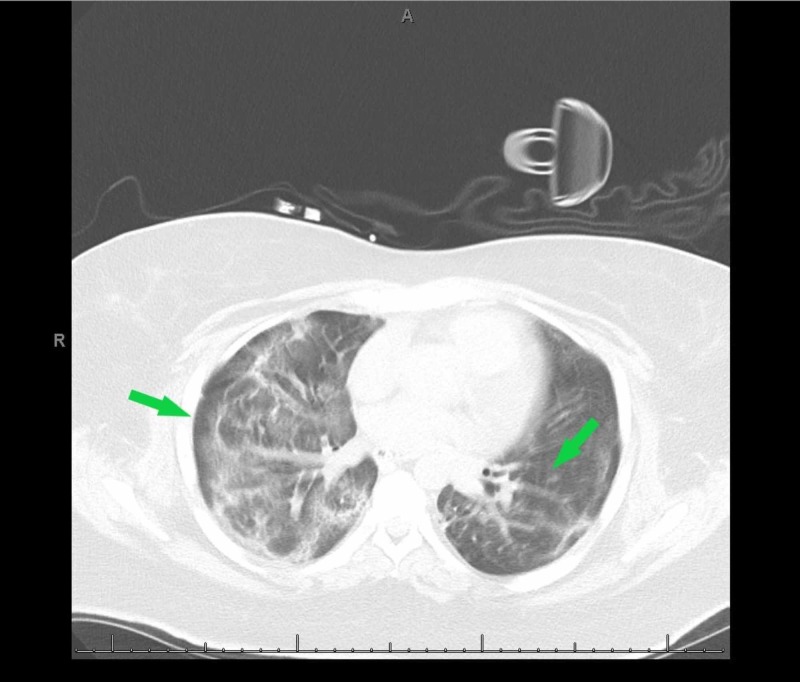
A computed tomography (CT) of the chest revealing long opacities in bilateral lungs with superimposed intralobular and interlobular septal thickening (arrows) consistent with acute respiratory distress syndrome (ARDS)

A chest computed tomography (CT) was consistent with acute respiratory distress syndrome (ARDS) with partial pressure of oxygen (PaO2)/fraction of inspired oxygen (FiO2) ratio dropping as low as 77.5 consistent with severe ARDS (Figure 2). Testing to detect SARS-COV-2 came back positive. She was started on a course of hydroxychloroquine and optimized to lung-protective ventilation. Initial COVID-19 labs were indicative of cytokine release syndrome, while a high A-a gradient concerning for a V/Q mismatch was consistent with COVID-19 phenotype L (Table 1).

**Table 1:** Laboratory and imaging investigations in COVID-19 pneumonia with subsequent development of a stroke

INVESTIGATION	RESULT
White blood cell (initial presentation)	9.9
Hemoglobin (initial presentation)	14.2
Platelet (initial presentation)	173
Blood Urea Nitrogen (initial presentation)	30
Sodium (initial presentation)	150
Potassium (initial presentation)	4.3
Creatinine (initial presentation)	1.5
Creatinine Kinase (initial presentation)	232 U/L
Ferritin (1^st ^draw)	251
C-Reactive Protein (CRP) (1^st^ draw)	38.09
Erythrocyte Sedimentation Rate (ESR) (1^st^ draw)	111
D-Dimer (1^st^ draw)	2.72
Interleukin 6 (IL-6) (1^st^ draw)	25
Computed Tomography (CT) of the head (first)	No acute intracranial bleed, midline shift or mass effect. Fluid in bilateral mastoid air cells and diffuse mucosal thickening in bilateral sphenoid sinus and posterior ethmoid air cells.
Computed Tomography (CT) of the head (second)	There is presence of an ill-defined hypodensity within right frontal lobe. Possibility of acute/ subacute infarct need to be ruled out. No acute bleed. Opacification of paranasal sinuses and fluid within mastoid air cells.
Transthoracic Echocardiogram (TTE)	The ejection fraction is 60% - 65%. The left ventricle is normal size. There is normal left ventricular wall thickness. Left ventricular diastolic dysfunction consistent with grade I impaired relaxation. There is mild tricuspid valve regurgitation.
Bilateral carotid ultrasound with duplex	Bilateral common carotid arteries (CCA), visualized internal carotid arteries (ICA) and external carotid arteries are normal in course and caliber. No significant stenosis seen. Color flow and waveform patterns are unremarkable. Antegrade flow seen in the both vertebral arteries. Velocities: 1. Right ICA: 71 cm/sec 2. Left ICA: 61 cm/sec. ICA/CCA ratio - Right: 0.59 and Left: 0.67
Ferritin (2^nd^ draw)	437
CRP (2^nd^ draw)	161.54
Lactate Dehydrogenase (2^nd^ draw)	409
D-Dimer (2^nd^ draw)	3.64
IL-6 (2^nd^ draw)	17
Lupus Anticoagulant	Not detected
Factor 5 Leiden mutation	Normal Genotype
Protein C Activity	122%
Protein S Activity	82%
Antithrombin III Activity	110%
Cardiolipin IgM and IgG	Absent
Beta 2 Micro globulin	2.1 mg/L
Prothrombin gene mutation	Normal Genotype

**Figure 3:**
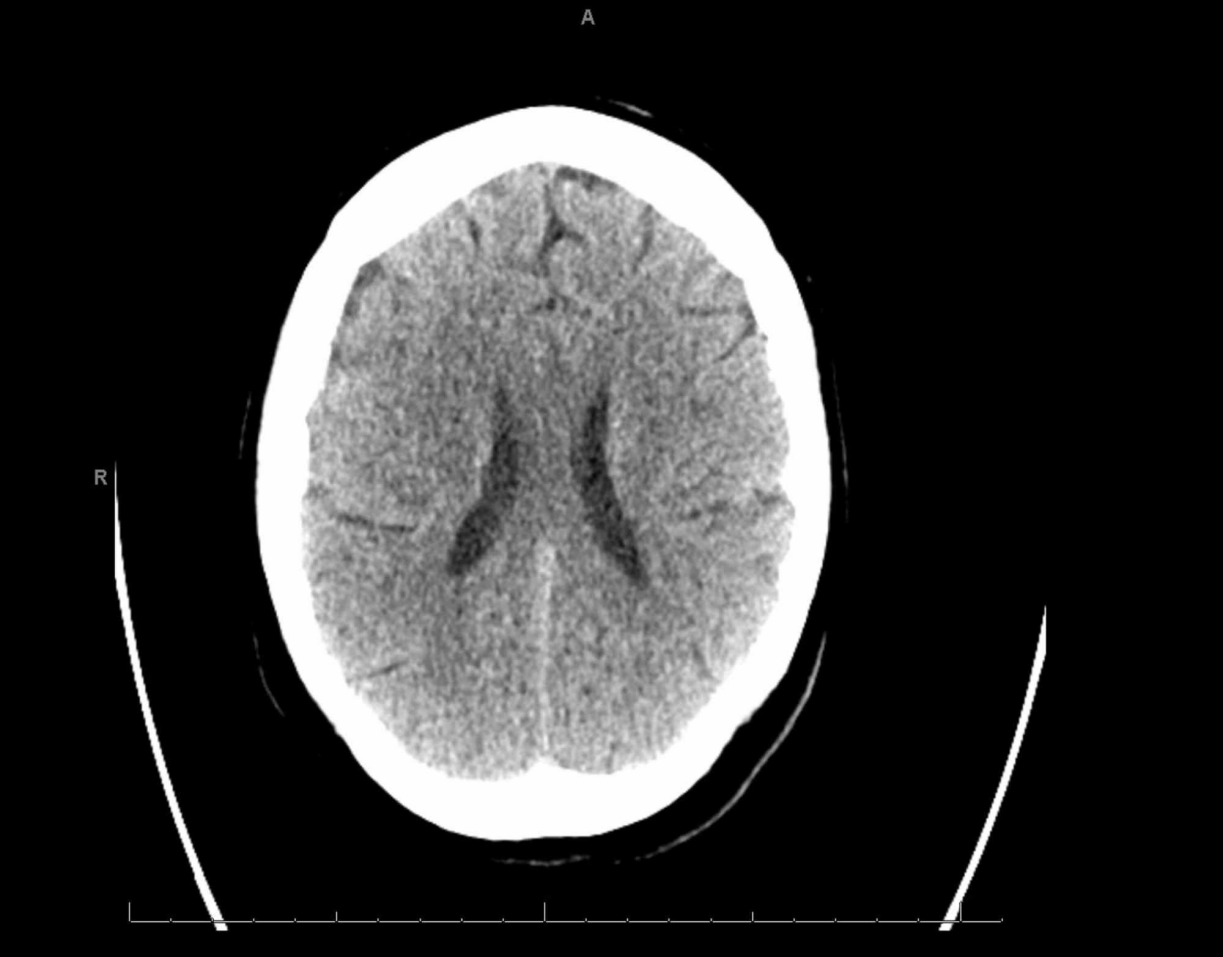
Non-contrast computed tomography (CT) of the head hours after symptom onset without signs of an acute infarct

**Figure 4:**
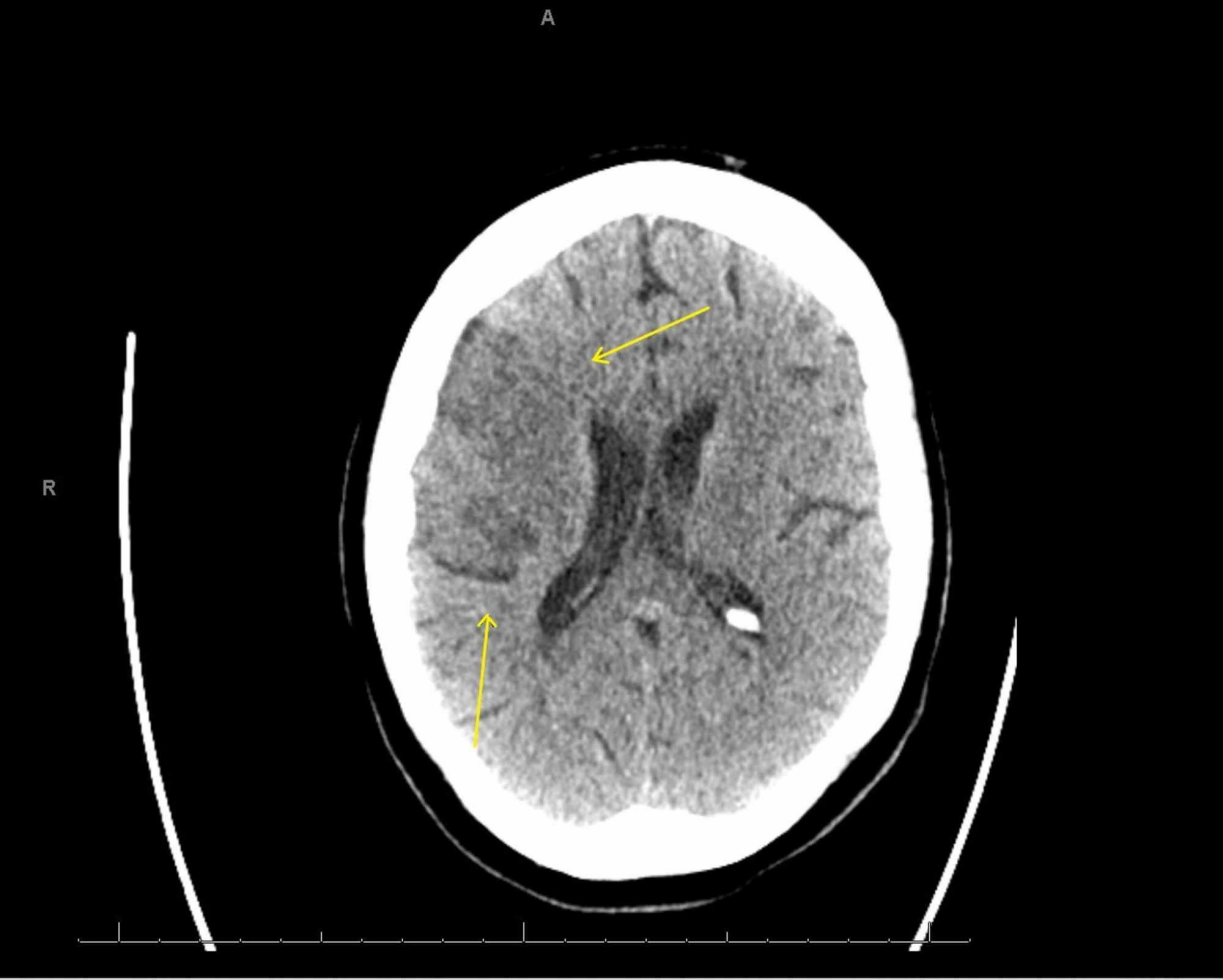
Non-contrast computed tomography (CT) of the head eight days after symptom onset significant for a new hypodensity in the right middle cerebral artery (MCA) distribution (arrows)

**Figure 5:**
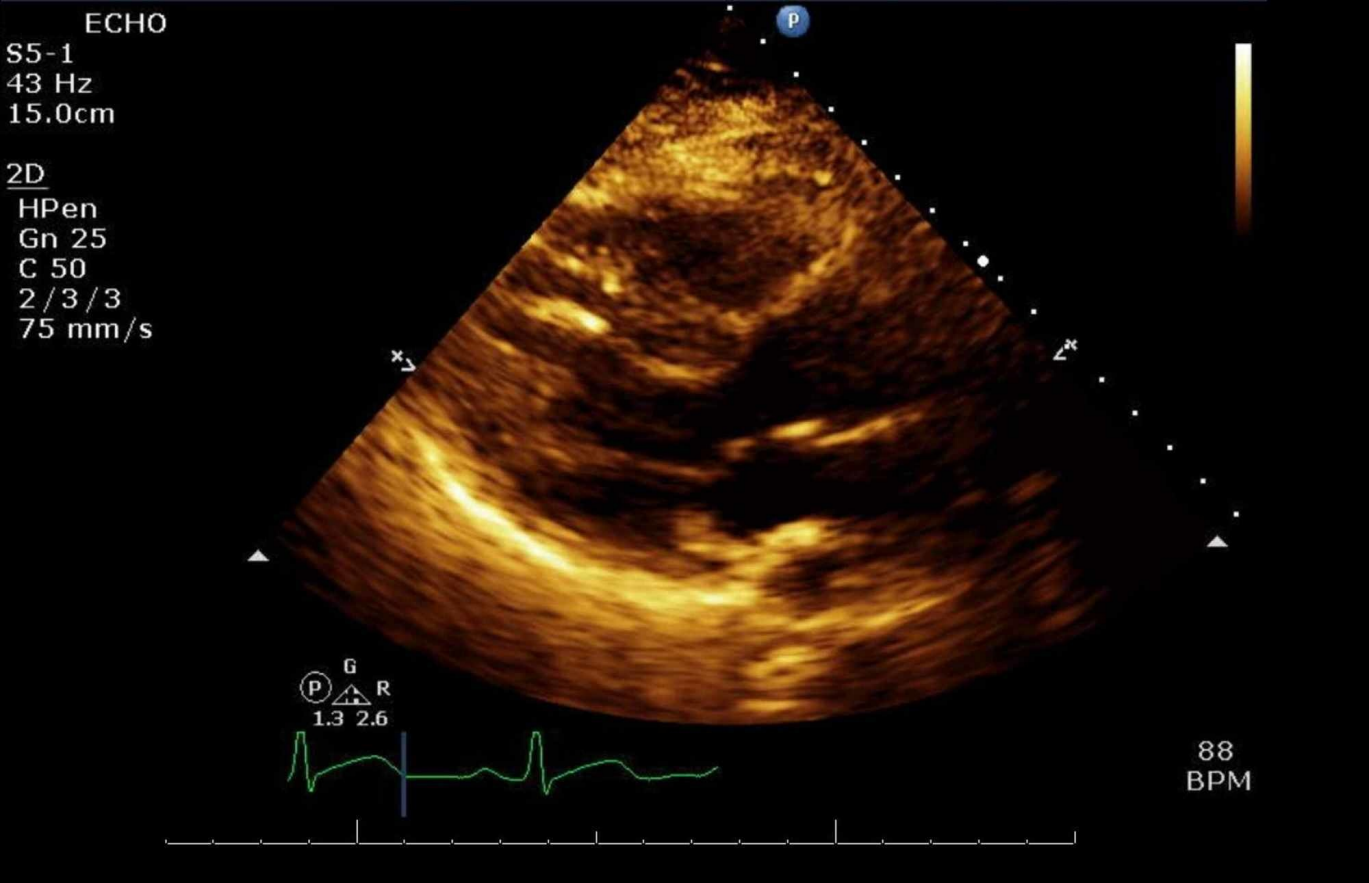
An unremarkable trans-thoracic echocardiogram (TTE)

She was placed on a short course of therapeutic anticoagulation. She was proned with intermittent paralytics to improve oxygenation and synchronize her to the ventilator. On daily awakening trials, she displayed significant features of confusion and encephalopathy. During saturation and weaning of her paralytic, new left-hand flaccidity was noted. The finding was subtle, and the National Institutes of Health Stroke Scale (NIHSS) could not be assessed due to her inability to follow commands. However, brainstem reflexes were preserved with pupillary and cough reflexes to stimuli, the ability to trigger spontaneous breaths, and response to pain. Delays in obtaining an emergent CT scan of her head ensued due to her positivity for COVID-19 and an equivocal stroke-like presentation. After appropriate precautions were set up, the final read of the CT was unrevealing for an acute or subacute infarct or hemorrhage (Figure 3). At the same time, her last known well could not be gauged due to her level of sedation and fluctuating mentation, precluding reperfusion therapies. Atorvastatin and aspirin were started for secondary prevention, while COVID-19 labs were repeated to trend progression of the disease. A repeat CT scan revealed ischemic changes in the region supplied by the right middle cerebral artery (MCA) consistent with a cortical stroke (Figure 4). Follow up echocardiogram, and hypercoagulable workup was negative as seen in Table 1, including ultrasounds in the upper and lower extremities looking for deep vein thromboses (DVTs) to look for sources of thrombi (Figure 5). After remaining on mechanical ventilation and nasogastric (NG) feeding for a prolonged period, a tracheostomy and percutaneous endoscopic gastrostomy (PEG) tube were performed.

Over the next few days, improvements in her mentation were noted with opening and tracking with her eyes, and the ability to start following commands. Physical therapy assessments recorded low mobility scores and severe disability while continuing to demonstrate periods of confusion. She was improving globally with the ability to tolerate breathing trials and follow commands. Conferred an overall poor prognosis, she was discharged to a rehabilitation facility.

## Discussion

The COVID-19 infection is an ongoing global pandemic with a high morbidity and mortality rate. SARS-CoV-2 has the propensity to infect the central nervous system, with autopsy reports revealing hyperemia and edema in affected patients [3]. COVID-19 patients with cerebrovascular diseases were noted to be at a higher risk for critical illness and multiorgan dysfunction based on previous reports from the SARS epidemic [6]. Amongst retrospective studies looking at the development of neurologic features during critical COVID-19 illness, 3.4% - 5% were found to have new ischemic strokes, with the youngest patient at 55 years of age [5,7]. In our patient with no apparent risk factors for a CVA and a young age, this case demonstrates that patients, regardless of their age, are at risk for severe neurological complications from this disease. COVID-19 is known to induce states of hypercoagulability by deranging pathways of hemostasis seen on thromboelastography (TEG) [8]. The viral load contributes to coagulopathy and endothelial dysfunction, similar to patients that developed strokes associated with SARS [5].

In our patient, serial d-dimers rose in the days leading up to the stroke, indicating an acute state of hypercoagulability despite receiving therapeutic anticoagulation. She received a short course of therapeutic anticoagulation but was suboptimally given due to the lack of data guiding the use of anticoagulation in severely ill COVID-19 patients. Another explanation could be an exaggerated systemic inflammation or a "cytokine" storm, often a marker of severe disease manifested in our patient with increasing pro-thrombotic markers, including IL-6 levels. Blood cultures and echocardiography were unremarkable for signs of an infection, while carotid ultrasound with dopplers was negative for low flow states between the carotids. Studies looking at neurological manifestation in COVID-19 patients, signs were most commonly noted early in the disease course presenting with muscle symptoms and confusion, while d-dimer levels were higher compared to non-severe infections [3].

In a pooled analysis of the current literature, CVAs were associated with a significantly increased risk (2.5x) of a severe form of COVID-19 disease. Critical illness-related encephalopathy and cytokines have been implicated as components of severe neurological manifestations seen with COVID-19, another feature noted in our patient before the development of her stroke, signifying a potential pre-cursor symptom [7]. The American Society of Hematology (ASH) recommends that all COVID-19 patients receive pharmacologic thromboprophylaxis, while seriously ill, high risk, or patients with high d-dimer levels should undergo therapeutic doses due to concerns for microvascular thrombosis, although these recommendations are based on limited data [9].

Amongst care for patients that developed strokes, only a small range of entities across the world can maintain their full range of acute stroke services, with most having to reorganize with the allocation of resources, including beds towards critically ill COVID-19 patients. The delivery of crucial therapies such as endovascular services, intravenous thrombolysis, or carotid endarterectomies in acute stroke patients has been compromised, with delays increasing door to needle times [10,11]. Patients that develop strokes requiring hospitalization during the COVID-19 pandemic are at an increased risk for suboptimal outcomes [12]. Acute stroke management pathways have been delineated in COVID-19 patients keeping in mind practice patterns, resource allocation, and the presence of a large vessel occlusion (LVO). Patients with non-LVO ischemic strokes are recommended to transfer to an infectious disease ward or a dedicated closely monitoring neurological ward [11].

## Conclusions

SARS-CoV-2 is known to induce states of hypercoagulability placing critically ill patients at high risk for vascular complications. Limited knowledge of the sequelae of this infection and guidelines leaves isolated case reports to guide clinical decision making, especially in patients exhibiting neurological complications. Despite being a disease that is known to traditionally affect the elderly, emerging reports of seemingly low-risk patients developing strokes are being published and thromboprophylaxis must be considered in seriously ill, high risk, or patients with high d-dimer levels. Encephalopathy is a frequently cited neurological manifestation of COVID-19 disease and can present as a sentinel sign for evolving strokes. Patients that develop strokes are at risk for suboptimal outcomes due to the diversion of resources to manage COVID-19 patients, while the development of acute stroke management pathways can help address these practice patterns. Further research is needed to parse out a temporal relationship between COVID-19 patients and the incidence of strokes including long term outcomes.
